# Temperature Stable Cold Sintered (Bi_0.95_Li_0.05_)(V_0.9_Mo_0.1_)O_4_-Na_2_Mo_2_O_7_ Microwave Dielectric Composites

**DOI:** 10.3390/ma12091370

**Published:** 2019-04-27

**Authors:** Dawei Wang, Shiyu Zhang, Di Zhou, Kaixin Song, Antonio Feteira, Yiannis Vardaxoglou, Will Whittow, Darren Cadman, Ian M. Reaney

**Affiliations:** 1 Department of Materials Science and Engineering, University of Sheffield, Sheffield S1 3JD, UK; kxsong@hdu.edu.cn; 2 Wolfson School of Mechanical, Electrical and Manufacturing Engineering, Loughborough University, Loughborough LE11 3TU, UK; S.Zhang@lboro.ac.uk (S.Z.); J.C.Vardaxoglou@lboro.ac.uk (Y.V.); W.G.Whittow@lboro.ac.uk (W.W.); D.A.Cadman@lboro.ac.uk (D.C.); 3 Electronic Materials Research Laboratory, Key Laboratory of the Ministry of Education & International Center for Dielectric Research, Xi’an Jiaotong University, Xi’an 710049, Shaanxi, China; zhoudi1220@xjtu.edu.cn; 4 College of Electronics Information, Hangzhou Dianzi University, Hangzhou 310018, China; 5 Christian Doppler Laboratory for Advanced Ferroic Oxides, Sheffield Hallam University, Sheffield S1 1WB, UK; a.feteira@shu.ac.uk

**Keywords:** cold sintering process, microwave dielectric ceramics, graded radial index lens

## Abstract

Dense (Bi_0.95_Li_0.05_)(V_0.9_Mo_0.1_)O_4_-Na_2_Mo_2_O_7_ (100−x) wt.% (Bi_0.95_Li_0.05_)(V_0.9_Mo_0.1_)O_4_ (BLVMO)-x wt.% Na_2_Mo_2_O_7_ (NMO) composite ceramics were successfully fabricated through cold sintering at 150 °C under at 200 MPa for 30 min. X-ray diffraction, back-scattered scanning electron microscopy, and Raman spectroscopy not only corroborated the coexistence of BLVMO and NMO phases in all samples, but also the absence of parasitic phases and interdiffusion. With increasing NMO concentration, the relative pemittivity (*ε_r_*) and the Temperature Coefficient of resonant Frequency (TCF) decreased, whereas the Microwave Quality Factor (*Qf*) increased. Near-zero TCF was measured for BLVMO-20wt.%NMO composites which exhibited *ε_r_* ~ 40 and *Qf* ~ 4000 GHz. Finally, a dielectric Graded Radial INdex (GRIN) lens was simulated using the range of *ε_r_* in the BLVMO-NMO system, which predicted a 70% aperture efficiency at 26 GHz, ideal for 5G applications.

## 1. Introduction

Microwave (MW) dielectrics are used in wireless communication systems as resonators, filters, and capacitors [1]. For miniaturization and reliability, microwave devices are fabricated from Low/Ultra-Low Temperature Co-fired Ceramics (LTCC and ULTCC) due to their compatibility with sustainable and cheap electrodes such as Ag, Cu and Al [2,3,4,5,6]. Typically, MW ceramics have permittivity, 10 < *ε*_r_ < 100, and quality factor, 2000 < *Qf* < 200,000, depending on the precise application along with near-zero Temperature Coefficient of resonant Frequency (TCF < ±10 MK^−1^) [7,8,9,10,11,12]. Dielectric resonators require ultra-high *Qf* (>40,000 GHz) and medium permittivity (20 < ε_r_ < 50) whereas LTCC typically have low ε_r_ (~10) and require only moderate *Qf* (~2000) for 3/4G mobile technology [7,8,9,10,11,12].

Recently, the Cold Sintering Process (CSP) has shown potential to densify ceramics/composites/devices at <200 °C [13,14,15,16,17,18,19,20,21,22,23]. Kahari et al., densified Li_2_MoO_4_ (LMO) ceramics at room temperature by adding water and applying pressure to powders. CSP LMO ceramics exhibited *ε*_r_ and *Qf* similar to conventional sintering [13]. Subsequently, CSP was studied by Guo et al., who applied this densification method to many different microwave materials and devices, including MoO_3_, LMO, Na_2_Mo_2_O_7_, K_2_Mo_2_O_7_, (LiBi)_0.5_MoO_4_ and Na_2_Mo_2_O_7_ (NMO)-xPTFE composites [17,18,19,20,21,22,23,24]. More recently, Hong et al. investigated the plastic deformation and densification of NaCl at room temperature [20], and Induja et al. densified Al_2_SiO_5_ ceramics using CSP with the addition of NaCl [21]. Our recent work has demonstrated that low TCF (−4.7 ppm/°C) and high *Qf* (16,000–22,000 GHz) could be achieved in Na_0.5_Bi_0.5_MoO_4_-Li_2_MoO_4_ and magnetodielectric Li_2_MoO_4_-BaFe_12_O_19_ composites, respectively [22,23,24].

Among reported CSP microwave materials, only Na_0.5_Bi_0.5_MoO_4_-Li_2_MoO_4_ composites have been shown to have near zero TCF but with a comparatively low *ε*_r_ (17.4) [22]. In the present work, (Bi_0.95_Li_0.05_)(V_0.9_Mo_0.1_)O_4_ (BLVMO, *ε*_r_ = 76, TCF = +81 ppm/°C) and Na_2_Mo_2_O_7_ (NMO, *ε*_r_ of 11.6, TCF of −99 ppm/°C) were selected as cold sintering end-members to fabricate a composite series with the anticipation for delivering a medium ε_r_ (*ca.* 40–50), zero TCF ceramic suitable for MW applications [25,26,27,28]. The potential use CSP composites in a novel graded radial index (GRIN) dielectric lens is discussed.

## 2. Experimental Section

BLVMO and NMO powders were synthesized separately by solid-state reaction. Raw materials, including V_2_O_5_ (>99%, Acros Organics, Fisher Scientific, Waltham, MA, USA), MoO_3_ (>99%, Acros Organics), Na_2_CO_3_ (99.9%, Fisher Scientific, Waltham, MA, USA), Li_2_CO_3_ (99.9%, Sigma-Aldrich, St. Louis, MO, USA) and Bi_2_O_3_ (99.9%, Acros Organics) were batched and ball-milled in isopropanol for 4 h. Dried powders were calcined at 600 °C and 500 °C for BLVMO and NMO, respectively. To prepare (100−x) wt.% BLVMO-x wt.% NMO (x = 0, 5, 10, 20, 40, 50, 80, 100) composite ceramics, BLVMO and NMO powder was mixed with 5–10 wt.% deionized water. Mixtures were hot-pressed 30 min at 150 °C at 200 MPa and dried 24 h at 120 °C to remove residual moisture. In addition, BLVMO and NMO bulk ceramics were conventionally sintered at 690 and 610 °C, respectively.

Bulk densities of ceramic pellets were calculated by the geometric method. Crystal structure, phase assemblage, microstructures of ceramic pellets were characterised by X-ray powder diffraction (XRD, D2 Phaser, Bruker, Billerica, MA, USA) using Cu*Kα* radiation, scanning electron microscopy (SEM, Inspect F, FEI, Hillsboro, OR, USA) and Raman spectroscopy (inVia Raman microscope, Renishaw, Wotton-under-Edge, UK) using a green laser with 514.5 nm at room temperature, respectively. Microwave properties of ceramic pellets were determined by a TE_01δ_ dielectric resonator method using a vector network analyzer (R3767CH, Advantest Corporation, Tokyo, Japan). A Peltier device heated the cavity to measure the resonant frequency (*f*) from 25 °C to 85 °C. TCF was calculated according to:

(1) $$\mathrm{TCF}=\;\frac{f_{T}-f_{T_{0}}}{f_{T_{0}}\times \left(T-T_{0}\right)}\;\times 10^{6}$$

where the $f_{T}$ and $f_{T_{0}}$ were the TE_01__δ_ resonant frequencies at temperature *T* and *T_0_*, respectively.

## 3. Results and Discussion

**Figure 1:**
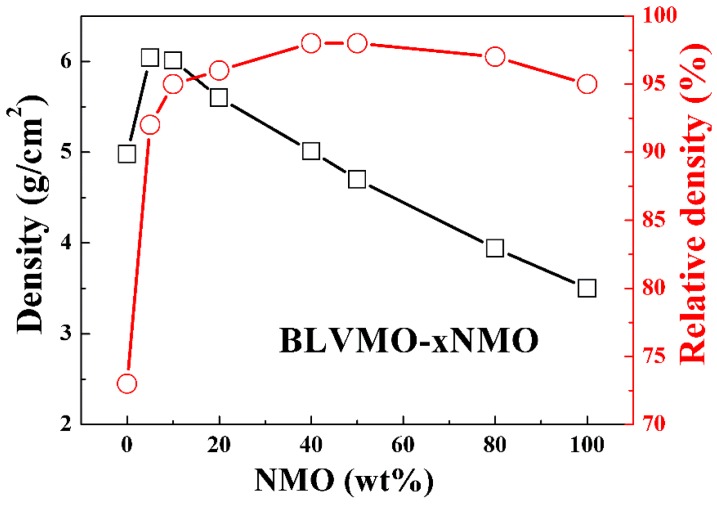
Bulk and relative densities of (100−x) wt.% (Bi_0.95_Li_0.05_)(V_0.9_Mo_0.1_)O_4_ (BLVMO)-x wt.% Na_2_Mo_2_O_7_ (NMO) ceramic composites.

The bulk and relative densities of CSP BLVMO are 4.98 g/cm^3^ and 73%, respectively, which increase to 6.04 g/cm^3^ and 98% with the addition of NMO (Figure 1 and Table 1). Following an initial increase for x = 0.05, bulk densities decreased linearly due to a lower theoretical density of NMO compared with BLVMO (6.85 g/cm^3^ and 3.69 g/cm^3^ for BLVMO and NMO, respectively) [25,26,27,28]. The relative densities of (100−x) wt.% BLVMO-x wt.% NMO ceramics are >90% (except pure BLVMO), attaining 98% for 40 wt.% NMO, confirming that dense (100−x) wt.% BLVMO-x wt.% NMO composites could be readily fabricated by CSP. 

**Figure 2:**
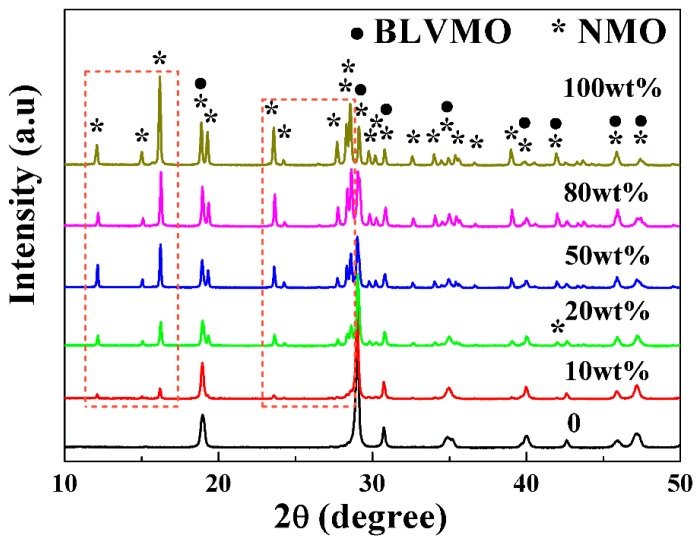
X-ray diffraction (XRD) patterns of (100−x) wt.% BLVMO-x wt.% NMO ceramic composites.

Room-temperature XRD patterns of CSP BLVMO, NMO and (100−x) wt.% BLVMO-x wt.% NMO samples in the 10°–50° 2*θ* range are shown in Figure 2. BLVMO has a tetragonal scheelite structure (PDF 48-0744) [26,27,28], with no evidence of splitting of main diffraction peaks. NMO has an orthorhombic structure with symmetry described by the space group *Cmca* (PDF 01-073-1797, *a* = 7.164 Å, *b* = 11.837 Å, *c* = 14.713 Å, *Z* = 8) [25]. All reflections in the XRD data for BLVMO-NMO ceramic composites can be ascribed to BLVMO and NMO and the intensity of NMO diffraction peaks increases with the concentration of NMO, as marked in Figure 2. Coexistence of peaks corresponding to BLVMO and NMO appear in all compositions with 0 < x < 1, and there is no apparent shift in peak position, indicating no interaction between these two end-members. 

**Figure 3:**
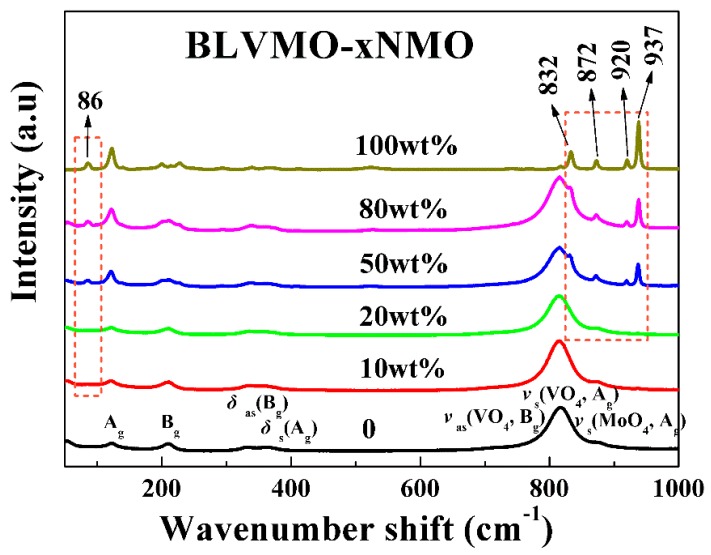
Raman spectra of (100−x) wt.% BLVMO-x wt.% NMO ceramic composites.

Room-temperature Raman spectra of CSP BLVMO, NMO and (100−x) wt.% BLVMO-x wt.% NMO ceramics are shown in Figure 3. According to group theory and irreducible representations, there are 15 and 129 different vibrational modes in BLVMO and NMO [26,27,28,29], respectively, given as follows:

*Γ*_BLVMO_ = 3A_g_ + 2A_u_ + 6B_g_ + 4B_u_ (2)

*Γ*_NMO_ = 18A_g_ + 13A_u_ + 15B_1g_ + 19B_1u_ + 14B_2g_ + 18B_2u_ + 19B_3g_ + 13B_3u_ (3)

In BLVMO, nine 3A_g_ + 6B_g_ modes are Raman active and six 2A_u_ + 4B_u_ modes are IR active [26,27,28]. In NMO, translations of Na and Mo atoms give 3A_g_ + 2A_u_ + 3B_1g_ + 4B_1u_ + 3B_2g_ + 4B_2u_ + 3B_3g_ + 2B_3u_ and 3A_g_ + 2A_u_ + 3B_1g_ + 4B_1u_ + 2B_2g_ + 3B_2u_ + 4B_3g_ + 3B_3u_ modes, respectively. Three B_1u_ + B_2u_ + B_3u_ modes are acoustic active and the remaining 12A_g_ + 9A_u_ + 9B_1g_ + 12B_1u_ + 9B_2g_ + 12B_2u_ + 19B_3g_ + 9B_3u_ modes correspond to stretching and bending modes of MoO_4_ and MoO_6_ octahedra [29]. The Raman spectra of (100−x) wt.% BLVMO-x wt.% NMO composites consist of a superposition of the spectral features exhibited by each individual phase, further confirming the coexistence of BLVMO and NMO in composite ceramics. Furthermore, the intensity of the NMO Raman modes increases with increasing NMO concentration. Several Raman bands in NMO (~86, 832, 872, 920 and 937 cm^−1^) are visible in all (100−x) wt.% BLVMO-x wt.% NMO compositions, confirming the coexistence of BLVMO and NMO in the composites.

**Figure 4:**
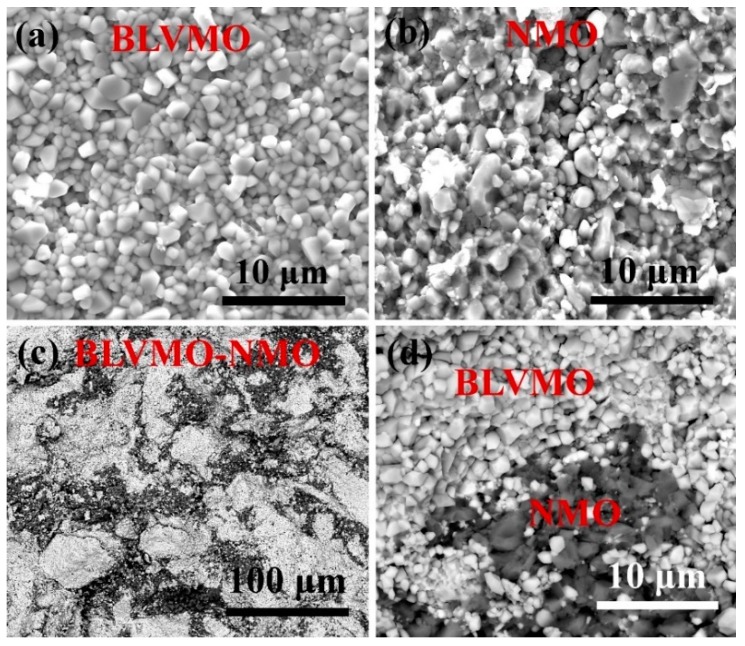
The SEM and BSE images of (**a**) conventionally-sintered BLVMO, (**b**) cold-sintered NMO, and (**c**,**d**) cold-sintered BLVMO-20 wt.% NMO samples.

Back-Scattered Electron (BSE) scanning electron microscope images of fracture surfaces of conventionally-sintered BLVMO, cold-sintered BLVMO-20wt.%NMO and NMO are revealed in Figure 4. Dense microstructures are visible in all three compositions, in agreement with the data presented in Figure 1 and Table 1. The average grain size of BLVMO (1–2 μm, Figure 4a) is smaller than that of NMO (2–5 μm, Figure 4b), consistent with previous reports [25,26,27,28]. Figure 4c,d shows the composites to be composed two chemically distinct and discrete phases with EDS confirming the dark and light contrast to be NMO and BLVMO, respectively, in agreement with XRD and Raman (Figure 2 and Figure 3). 

**Figure 5:**
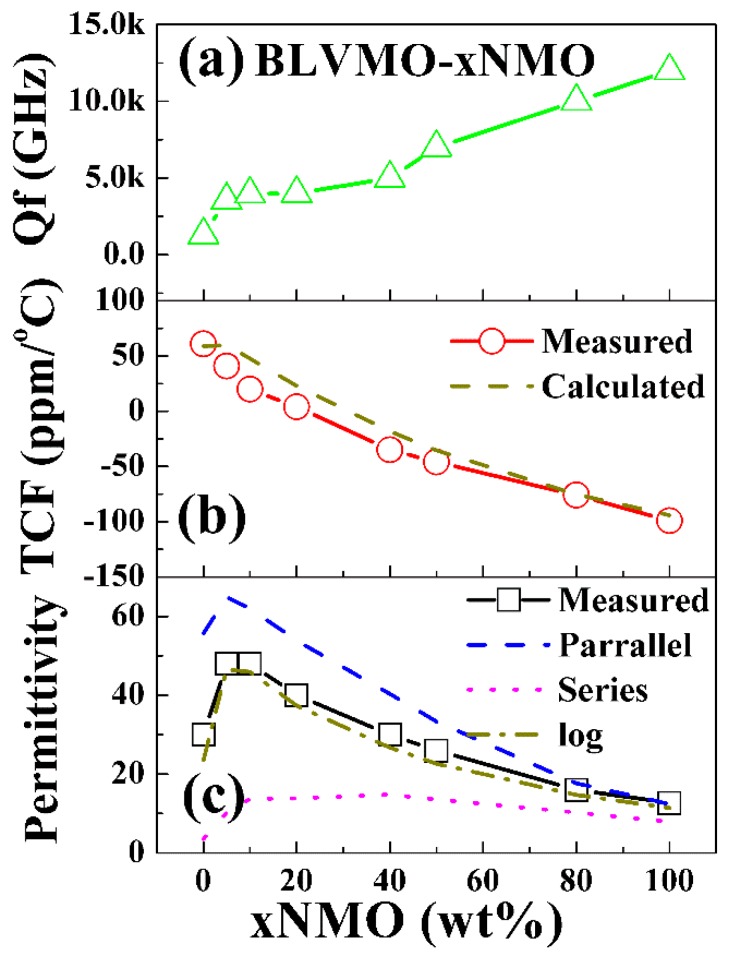
The microwave properties of (100−x) wt.% BLVMO-x wt.% NMO ceramic composites as a function of x (NMO fraction). (**a**) *Qf*, (**b**) TCF, (**c**) *ε*_r_.

The microwave properties of (100−x) wt.% BLVMO-x wt.% NMO as a function x are presented in Figure 5 and also listed in Table I. Low relative density (73%) of CSP BLVMO is observed which gives rise to lower *ε*_r_ (30) and *Qf* (1300 GHz) than for conventionally-sintered BLVMO, Table 1. *ε*_r_ and TCF values decrease linearly from 48 and +41 ppm/°C, respectively, for BLVMO-5 wt.%NMO to 12.7 and −99 ppm/°C for NMO. Near-zero TCF (−4 ppm/°C) is obtained for BLVMO-20 wt.%NMO. *Qf* increases from 1300 GHz for BLVMO to 12,000 GHz for NMO, as shown in Figure 5 and Table 1.

Provided there are no chemical reactions between phases, the *ε*_r_ in composites may be predicted by different mixing laws, as follows [22]:

parallel mixing law, *ε* = *V*_1_*ε*_1_ + *V*_2_*ε*_2_ + *V*_0_*ε*_0_ (4)

series mixing law, 1/*ε* = *V*_1_*/ε*_1_ + *V*_2_*/ε*_2_ + *V*_0_*/ε*_0_ (5)

(6) $$\mathrm{logarithmic}\;\mathrm{mixing}\;\mathrm{law},\;\varepsilon =\;\varepsilon_{1}^{V_{1}}\varepsilon_{2}^{V_{2}}\varepsilon_{0}^{V_{0}}\;i.e.,\;\mathrm{lg}\varepsilon \;=\;V_{1}\mathrm{lg}\varepsilon 1_{\;}+\;V_{2}\mathrm{lg}\varepsilon 2_{\;}+\;V_{0}\mathrm{lg}\varepsilon_{0}$$

where *ε*_1_, *ε*_2_ and *ε*_0_ are the *ε*_r_ of phase 1, phase 2 and air, respectively and *V*_1_, *V*_2_ and *V*_0_ (*V*_1_ + *V*_2_ + *V*_0_ = 1) are their respective volume fractions. As shown in Figure 5, *ε*_r_ for (100−x) wt.% BLVMO-x wt.% NMO composite ceramics is within the range of calculated values for Equations (4) and (5), and close to the values obtained using Equation (6), indicating that *ε*_r_ follows a logarithmic mixing law with x. TCF of composites is predicted with a simple mixing rule, which is derived from the Equation (6) [30]:

TCF = *V*_1_TCF_1_ + *V*_2_TCF_2_ (7)

where TCF_1_ and TCF_2_ correspond to the TCF of the two phases. TCF is consistent with calculated values using Equations (7), as shown in Figure 5b, suggesting they can be predicted using simple rules of mixture.

Microwave dielectric properties of various cold-sintered microwave dielectric materials are compared in Table 2. Numerous materials (*ρ_r_* = 83.7%–100%) with a range of dielectric properties (2.1 ≤ *ε*_r_ ≤ 48, 2240 ≤ *Qf* ≤ 135,700 GHz, −174 ≤ TCF ≤ 184 ppm/°C) can be densified, indicating that CSP is an effective, and energy-saving strategy for the fabrication of microwave devices [31,32]. (100−x) wt.% BLVMO-x wt.% NMO (x = 10–20) exhibits the highest value of *ε_r_* (~48) for near-zero TCFs cold-sintered microwave dielectric materials and is thus attractive for RF applications.

The low sintering temperature and absence of lateral shrinkage suggest that (100−x) wt.% BLVMO-x wt.% NMO composites have the potential for many novel RF applications including antennas, temperature stable capacitors, LTCC substrates and GRaded INdex (GRIN) dielectric lenses.

**Figure 6:**
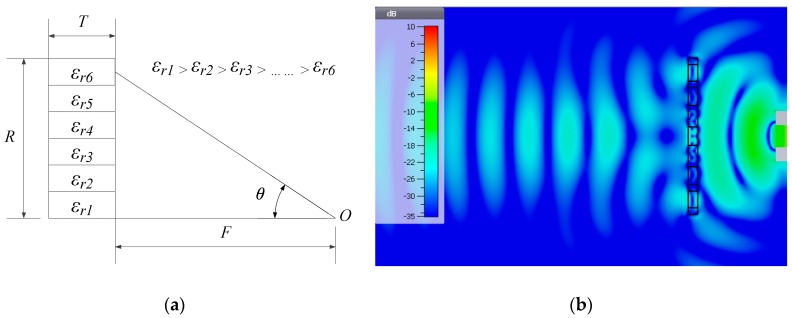
(**a**) Lens design principle; (**b**) Simulated electric field of a ceramic Graded Radial INdex (GRIN) lens that transforming spherical wavefronts into a planar wavefront at 26 GHz.

A GRIN lens is an antenna component for transforming a spherical to a planar wavefront, and enables highly directive antennas and shaped beams. A lightweight, flat lens may be used in the proximity of the feed to realise a compact system that is desired by 5G applications. For practical fabrication, the index profile of a flat lens is usually graded to several tight-fitted rings with radially reduced *ε_r_*. GRIN lenses may be fabricated from concentric dielectric cylindrical rings with graded *ε*_r_, Figure 6a. The simulated electric field of a ceramic GRIN lens is displayed in Figure 6b, transforming a spherical to a planar wavefront at 26 GHz.

The design parameters of a lens are shown in Table 3 and Table 4. The dielectric lens is comprised of six concentric rings; the outermost has the lowest effective *ε_r_* (12.7), while the centre has the highest *ε_r_* (48). The high *ε_r_* ceramic reduces the thickness of the lens (miniaturises) compared with low *ε_r_* materials such as polymers.

**Figure 7:**
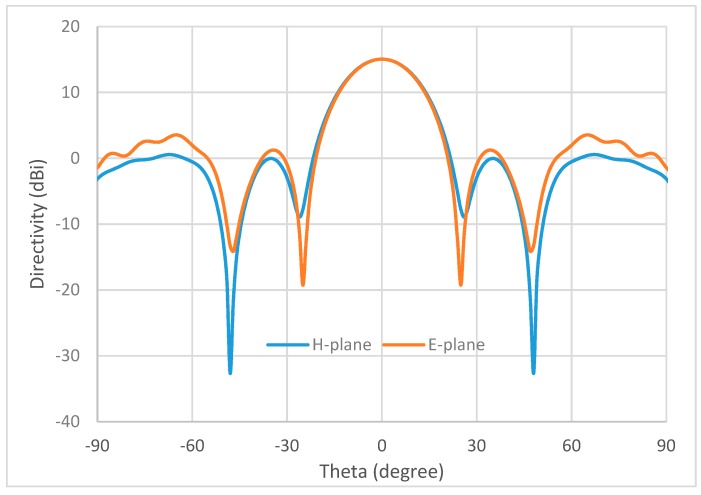
Simulated far-field radiation patterns of the ceramic GRIN lens at 26 GHz.

Lens performance was simulated using CST Microwave Studio. An open-ended *Ka*-band waveguide (7.112 mm × 3.556 mm) was used to illuminate the lens. The boresight directivity is increased across the whole frequency range from 26 to 40 GHz. The relative increase compared to the case with no lens is between 4.6 and 8.5 dB. The aperture efficiency of the lens is ~70% at 26 GHz. The simulated E-plane (i.e., the plane containing the electric field vector) and H-plane (the plane containing the magnetic field vector, normal to the E-plane) radiation patterns of the lens are illustrated in Figure 7.

## 4. Conclusions

The (100−x) wt.% BLVMO-x wt.% NMO ceramics with relative density of 92%–98% were fabricated by cold sintering process at 150 °C/30 min/200 MPa. No evidence of chemical interaction was observed in composites, except BLVMO and NMO phases, by means of SEM, XRD and Raman spectroscopy. As x increased, TCF and *ε*_r_ decreased, while *Qf* increased. Near-zero TCF ~ +4 ppm/°C was measured for BLVMO-20wt%NMO with *ε_r_* ~ 40 and *Qf* ~ 4000 GHz. A dielectric GRIN lens was designed and simulated exhibiting 70% aperture efficiency at 26 GHz, which we propose may be fabricated using (100−x) wt.% BLVMO-x wt.% NMO composites.

## Figures and Tables

**Table 1:** Sintering temperatures (ST), relative densities (*ρ_r_*), and microwave dielectric properties of BLVMO, NMO and (100−x) wt.% BLVMO-x wt.% NMO ceramics.

Composition	ST (°C)	*ρ_r_* (%)	*ε_r_*	tan*δ* (%)	*Qf* (GHz)	TCF (ppm/°C)
BLVMO	150	73	30	0.003	1300	+61
5% NMO	150	92	48	0.0014	3565	+41
10% NMO	150	95	48	0.0012	3959	+20
20% NMO	150	96	40	0.0012	4000	+4
40% NMO	150	98	30	0.001	5000	−35
50% NMO	150	98	26	0.001	7000	−46
80% NMO	150	97	16	0.0007	10000	−76
NMO	150	95	12.7	0.0005	12000	−99
BLVMO	690	96	76	0.0006	7000	+81
NMO	610	87	11.6	0.0005	19000	−78

**Table 2:** Comparison of relative densities, and microwave properties of cold-sintered microwave dielectric materials (* unpublished work, *ρ_r_* = relative density, PTFE = Polytetrafluoroethylene, LMO = Li_2_MoO_4_, BF12 = BaFe_12_O_19_, NBMO = Na_0.5_Bi_0.5_MoO_4_, BLVMO = (Bi_0.95_Li_0.05_)(V_0.9_Mo_0.1_)O_4_, NMO = Na_2_Mo_2_O_7_).

Compound	*ρ_r_* (%)	*ε_r_*	*Qf* (GHz)	TCF (ppm/^o^C)	Reference
PTFE	100	2.12	135,700	+60	*
Polystyrene	100	2.53	24,320	−5	*
Al_2_SiO_5_-NaCl	/	4.52	22,350	−24	[21]
KCl	98	4.74	7738	−149	*
LMO	95.5	5.1–5.61	10,200-30,500	−170	[13,14,15,16,17,18,19,22]
NaCl	97–99	5.22–5.55	12,000-49,600	−100	[20,21]
LMO-15%BF12	94.1	5.8	17,430	-	[23]
K_2_MoO_4_	100	6.37	26,500	−70	*
AgNaMoO_4_	90.8	9.3	7078	−120	*
K_2_Mo_2_O_7_	94.1–96	9.35–9.8	12,000–16,000	−63	[17], *
MoO_3_	83.7	9.91	11,800	−39	[24]
Na_2_Mo_2_O_7_	93.7–95	12.7–13.4	12,000–14,900	−99	[17], *
NBMO-20%LMO	93.6	17.4	7470	−4.7	[22]
NBMO-10%LMO	92.6	24.1	2240	+15	[22]
(LiBi)_0.5_MoO_4_	88	33.7–37	1700–2300	+180	[18]
BLVMO-20%NMO	96	40	4000	+4	this work
BLVMO-10%NMO	95	48	3959	+20	this work

**Table 3:** Designed parameters of a 3D-printed lens.

Parameter	Value
Diameter	*R* = 12.5 mm
Focal length	*F* = 12.5 mm
Thickness	*T* = 1.53 mm

**Table 4:** Dielectric constant values of the concentric dielectric rings.

Ring No.	*ε_r_*	Ring Outer Radius(mm)
1	48	1.1
2	40	4.9
3	30	7.8
4	26	8.8
5	16	11.5
6	12.7	12.5
